# Multiple Precursor Proteins of Thanatin Isoforms, an Antimicrobial Peptide Associated With the Gut Symbiont of *Riptortus pedestris*

**DOI:** 10.3389/fmicb.2021.796548

**Published:** 2022-01-05

**Authors:** Junbeom Lee, Wook Hyun Cha, Dae-Weon Lee

**Affiliations:** ^1^Metabolomics Research Center for Functional Materials, Kyungsung University, Busan, South Korea; ^2^Department of Bio-Safety, Kyungsung University, Busan, South Korea

**Keywords:** *Riptortus pedestris*, *Burkholderia insecticola*, symbiosis, thanatin isoforms, multiple precursor proteins

## Abstract

Thanatin is an antimicrobial peptide (AMP) generated by insects for defense against bacterial infections. In the present study, we performed cDNA cloning of thanatin and found the presence of multiple precursor proteins from the bean bug, *Riptortus pedestris*. The cDNA sequences encoded 38 precursor proteins, generating 13 thanatin isoforms. In the phylogenetic analysis, thanatin isoforms were categorized into two groups based on the presence of the membrane attack complex/perforin (MACPF) domain. In insect-bacterial symbiosis, specific substances are produced by the immune system of the host insect and are known to modulate the symbiont’s population. Therefore, to determine the biological function of thanatin isoforms in symbiosis, the expression levels of three AMP genes were compared between aposymbiotic insects and symbiotic *R. pedestris*. The expression levels of the *thanatin* genes were significantly increased in the M4 crypt, a symbiotic organ, of symbiotic insects upon systemic bacterial injection. Further, synthetic thanatin isoforms exhibited antibacterial activity against gut-colonized *Burkholderia* symbionts rather than *in vitro*-cultured *Burkholderia* cells. Interestingly, the suppression of *thanatin* genes significantly increased the population of *Burkholderia* gut symbionts in the M4 crypt under systemic *Escherichia coli* K12 injection. Overgrown *Burkholderia* gut symbionts were observed in the hemolymph of host insects and exhibited insecticidal activity. Taken together, these results suggest that thanatin of *R. pedestris* is a host-derived symbiotic factor and an AMP that controls the population of gut-colonized *Burkholderia* symbionts.

## Introduction

The bean bug, *Riptortus pedestris* (Hemiptera: Alydidae), possesses a specialized symbiotic organ in the posterior midgut region (named M4 crypt), where numerous crypts harbor specific gut symbionts, *Burkholderia insecticola*, recently reassigned to the genus *Caballeronia* (Kikuchi et al., 2005, 2007, 2011). These symbionts are orally acquired by *R. pedestris* nymphs from the environment of every generation, and are easily cultivated and genetically manipulated (Kim et al., 2013b,c, 2014, 2016b,^2017^; Lee et al., 2015). Based on these physiological characteristics, the *Riptortus-Burkholderia* symbiosis system has been recognized as a promising experimental model for studying insect-microbe interaction at the molecular and biochemical levels (Kim et al., 2016a). Notably, this model has several experimental advantages for studying insect symbiosis. By controlling the oral infection of *Burkholderia* cells, we can generate *Burkholderia* gut symbiont-colonized insects (Sym-insect) and gut symbiont-non-colonized insects (Apo-insect) in the laboratory (Kikuchi et al., 2007; Kim et al., 2015a; Lee J. B. et al., 2017). Additionally, as a large number of naïve *Burkholderia* gut symbionts can be collected from M4 crypts, it is feasible to study biochemical differences between *in vitro*-cultured *Burkholderia* cells and *in vivo*-colonized symbiotic cells (Kim et al., 2013c,2015b; Byeon et al., 2015). Accordingly, we recently reported unexpected observations and extended our understanding of how this symbiont interacts with host insects at the molecular and biochemical levels (Lee et al., 2019, 2020).

Insects have an immune system that comprises cellular and humoral immune responses (Hoffmann, 2003). When entomopathogenic microorganism infection occurs, the cellular immune response is activated, and the pathogen is immediately eliminated by phagocytosis, encapsulation, and nodulation (Lavine and Strand, 2002; Strand, 2008). At the same time, the humoral immune response of the insect is activated sequentially, and as a result, antimicrobial peptides (AMPs) are produced in the insect’s fat body tissue and secreted into the hemolymph to eradicate the infective pathogens (Lemaitre et al., 1997; Vallet-Gely et al., 2008). However, this type of immune response has been extensively studied in holometabolous insects (complete metamorphic), but not in hemimetabolous insects (incomplete metamorphic) (Kanost and Gorman, 2008; Hillyer, 2016; Yan and Hillyer, 2020).

For *R. pedestris* insect, we isolated and identified three types of AMPs, riptocin, defensin, and thanatin isoforms, from the hemolymph. These AMPs were found to exhibit effective antimicrobial activity against invasive pathogens (Kim et al., 2015b). In addition, we successfully purified a peptide trialysin from the salivary glands of *R. pedestris* to confirm the immune response in the oral infection route. Of note, this protein has been reported to play a crucial role in distinguishing gut commensal symbionts from pathogens (Lee D. J. et al., 2017).

Among these AMPs, thanatin isoforms have only been found in the hemipteran insect, *Podisus maculiventris* (spined soldier bug), which belongs to the same order as *R. pedestris* (Fehlbaum et al., 1996). Because the thanatin peptide has no sequence homology with other insect defense molecules, its exact role and significance in insects have not been elucidated. In previous study, we found that thanatin isoforms of *R. pedestris* were synthesized in the midgut region in which the gut symbiont resided (Park et al., 2018). However, we did not demonstrate why *R. pedestris* insects should control their gut bacterial populations with thanatin isoforms under infection. Therefore, we conducted a study to suggest the exact role of thanatin isoforms in the *R. pedestris*–*Burkholderia* symbiosis model. Based on these results, we hypothesized that thanatin isoforms can be unique AMPs that play an important role in hemimetabolous insects and symbiotic relationships. As the gut immune system of *R. pedestris* in a symbiotic condition has not been clearly elucidated, in this study, we investigated the gut immune response of host insects associated with thanatin isoforms and demonstrated the effect of this AMP on gut symbionts. Here, we found that the expression levels of only *thanatin* genes were increased in the M4 crypt of Sym-male insects compared to the two other AMPs tested upon systemic bacterial injection. Further, we revealed that thanatin isoforms not only function as AMPs, but also modulate the population of gut symbionts.

## Materials and Methods

### Bacterial Strains and Growth Conditions

The *Burkholderia insecticola* symbiont RPE75, a rifampicin-resistant mutant derived from the RPE64 strain (Kikuchi et al., 2011), was cultured until mid-log phase at 30°C in YG-RIF medium (0.5% yeast extract, 0.4% glucose, and 0.1% NaCl supplemented with 30 μg/ml rifampicin) (Lee J. B. et al., 2017). *Escherichia coli* K12, *Serratia marcescens* Db11, and *Staphylococcus aureus* RN4220 cells were cultured until mid-log phase at 37°C in Luria-Bertani medium (Gibco, United States) without any antibiotic substance (Kim et al., 2015a). All bacterial strains were incubated with vigorous shaking.

### Insect Rearing and *Burkholderia* Symbiont Infection

The bean bugs, *R. pedestris*, were reared in our insect laboratory at 28°C under a photoperiod of 16 h light and 8 h dark, as previously described (Lee et al., 2020). Nymphal insects were reared in clean plastic containers containing soybean seeds and distilled water containing 0.05% ascorbic acid (DWA). Upon reaching adulthood, the insects were transferred to larger containers (35 cm long, 35 cm wide, and 40 cm high); soybean seeds were added to the containers as food, and cotton pads were attached to the walls for egg laying. Eggs were collected daily and transferred to new cages for hatching. A cotton dish was soaked in *in vitro*-cultured *Burkholderia* (10^7^ cells/ml of DWA) was provided to the second instar nymphs for 12 h to generate *Burkholderia*-harboring Sym-insects (Lee et al., 2019). After the insects were fed the inoculum solution for 2 days, fresh DWA was provided instead of the inoculum solution.

### cDNA Cloning of Thanatin Isoforms From *Riptortus pedestris*

To obtain cDNA sequences of thanatin AMPs in the fat body, we used a Gene Racer kit (Invitrogen, United States) (Lee D. J. et al., 2017). After mRNA isolation from the fat body of 3-day-old *R. pedestris* male adults, truncated and non-mRNAs were removed with calf intestinal phosphatase by dephosphorylation of their 5′ phosphates according to the manufacturer’s instructions. The mRNA was then decapped with tobacco acid phosphatase. The ligated mRNA was reverse-transcribed using a GeneRacer oligo dT primer and Superscript III reverse transcriptase (50°C, 1 h). To obtain the 5′ ends, cDNA was amplified using a GeneRacer 5′ forward primer and a gene-specific 5′ reverse primer or a GeneRacer 5′ nested forward primer and a gene-specific 5′ nested reverse primer with Phusion High-Fidelity DNA polymerase (NEB, United States). The PCR conditions were as follows: 95°C for 30 s, followed by 35 cycles of 95°C for 30 s, 65°C for 30 s, and 68°C for 2 min, and finally 68°C for 5 min. To obtain the 3′ ends, the cDNAs were amplified using a GeneRacer 3′ reverse primer and a gene-specific 3′ forward primer or a GeneRacer 3′ nested reverse primer and a gene-specific 3′ nested forward primer. The PCR conditions were as follows: 95°C for 30 s, followed by 35 cycles of 95°C for 30 s, 65°C for 30 s, and 68°C for 2 min, and finally 68°C for 5 min. The primer sets used for RACE-PCR are listed in Table 1. To determine the nucleotide sequence of the target PCR products, PCR amplification mixtures were cloned using a TOPO TA cloning kit (Invitrogen, United States) and sequenced.

**TABLE 1:** Primer set used for RACE-PCR.

Name	Primer sequence (5′→3′)
**5′ ends**	
GeneRacer 5′_F	CGACTGGAGCACGAGGACACTGA
gene-specific 5′_R	CCGGTCTTCCTGTTGCAGTAAATTAT
GeneRacer 5′ nested_F	GGACACTGACATGGACTGAAGGAGTA
gene-specific 5′ nested_R	TTATCGGTACTCTCCCCCTTTTCTG
**3′ ends**	
GeneRacer 3′_R	GCTGTCAACGATACGCTACGTAACG
gene-specific 3′_F	ATGACTTCATCAAGATGCATGTTGGTG
GeneRacer 3′ nested_R	CGCTACGTAACGGCATGACAGTG
gene-specific 3′ nested_F	GCTAGCTTGCCTAGCTTGTATTGG

### Susceptibility Assay Against Thanatin Isoforms

*Escherichia coli* K12, *S. marcescens* Db11, *S. aureus* RN4220, and cultured-*Burkholderia* cells were cultured in LB and YG medium and washed with 10 mM phosphate buffer (PB; pH 7.0) to remove the medium components. Gut-colonized *Burkholderia* cells were collected from the fifth-instar nymphs of Sym-insects by dissection of the M4 crypt and washed twice with 10 mM PB to remove substances from *R. pedestris* (Byeon et al., 2015; Kim et al., 2015b). To test the antimicrobial activity of thanatin isoforms, the bacterial cells were suspended in 10 mM PB to 10^3^ cells/50 μl for each sample and subsequently incubated with 50 μl of synthesized thanatin solution (Anygen, South Korea) at concentrations of 0.2, 0.4, 1, 2, or 4 μg/ml for 2 h at 30°C. After incubation, the reaction mixtures were spread on LB or YG agar plates containing selective antibiotics. The colonies grown after 48 h of incubation at 30°C were counted.

### Thanatin Expression Upon Immune Challenging

qRT-PCR was performed as previously described (Kim et al., 2015a; Lee J. B. et al., 2017). Briefly, *E. coli* K12 and *S. marcescens* Db11 cells were adjusted to 1.0 × 10^7^ cells/ml in 10 mM PB. Two microliters of the bacterial cells were systemically injected into 3-day-old *R. pedestris* male adults for immune induction. After incubation at 28°C for 3 h, immune-challenged fat body and all five midgut regions were collected from the insects by dissection. Total RNA was isolated using TRIzol reagent (Invitrogen, United States), according to the manufacturer’s recommendations. Thereafter, 500 ng of total RNA was converted to cDNA using TOPscript RT DryMix containing oligo-dT primers (Enzynomics, South Korea). The synthesized cDNAs were diluted 20-fold, and qRT-PCR was performed on a QuantStudio™3 Real-Time PCR System (Thermo Fisher Scientific Inc., United States). The PCR cycling condition was as follows: 95°C for 10 min, followed by 40 cycles of 95°C for 10 s, 60°C for 15 s, and 72°C for 20 s. The primer sets used for qRT-PCR are listed in Table 2. The comparative *C*_*T*_ (ΔΔ*C*_*T*_) method was used to calculate the relative gene expression levels based on the elongation factor 1α gene (*EF1*α) of *R. pedestris* (GenBank accession #AB591382) as an endogenous control gene (Lee J. B. et al., 2017). All analyses were performed using the QuantStudio™ Design & Analysis Software Ver 1.5.2 (Thermo Fisher Scientific Inc., United States).

**TABLE 2:** Primer set used for qRT-PCR.

Name	Primer sequence (5′→3′)
Thanatin_F	ATCTTGCAGAACTCCAGCGC
Thanatin_R	CTGTTGCAGTAAATTATCGGTACT
Riptocin_F	TCCGAAGCTGAGGGTCTTCCCG
Riptocin_R	TCCGCATCCAAGTTCGCGTCC
Defensin_F	TCGGTCGGACTGAGACTGAA
Defensin_R	TTGCCGCCTTTGTATCCCTT
EF1α_F	CCTGCATCCGTTGCTTTTGT
EF1α_R	GGCATCGAGGGCTTCAATAA

### RNA Interference

RNA interference (RNA*i*) was performed as described previously (Lee J. B. et al., 2017). Double-stranded RNA (dsRNA) for silencing the *thanatin* genes (target) and the *ampicillin-resistant* gene (mock control) were synthesized using the primers listed in Table 3. Each recombinant plasmid was constructed by cloning the PCR products that were amplified from *R. pedestris* fat body cDNA with sense and antisense primers into the pT7Blue T-vector (Novagen, United States). Each T-cloned plasmid was amplified by PCR using a specific primer set containing the T7 promoter region. The cycling conditions were as follows: 95°C for 10 min, followed by 35 cycles of 95°C for 30 s, 65°C for 30 s, and 72°C for 1 min, and finally 72°C for 10 min. After purification of the product with a PCR purification kit (Enzynomics, South Korea), both sense and antisense strands of the transcript were simultaneously synthesized using the MEGA-script RNAi kit (Ambion, United States) with 500 ng of template DNA at 37°C for 2 h, according to the manufacturer’s instructions. The synthesized RNA product was treated with DNase I and RNase to remove the template DNA and ssRNA at 37°C for 30 min. The dsRNAs were purified using the MEGAclear kit (Ambion, United States) and quantified using a Nanodrop 2000 (Thermo Fisher Scientific, United States). The Sym-male insects were treated with dsRNA 200 ng/μl in DNase/RNase-free water using a glass capillary and an air-pressure injector (Picospritzer, Parker, United States). Briefly, two microliters of *thanatins* dsRNA or DNase/RNase-free water (for mock control) were systemically injected into the joint of the hind leg that connects to the thorax of the insect. The dsRNA-treated insects were reared in clean plastic containers, and colony-forming units (CFUs) in M4 crypts and hemolymph were measured on 4 days after dsRNA injection and 3 days after *E. coli* cells injection.

**TABLE 3:** Primer set used for RNA*i.*

Name	Primer sequence (5′→3′)
dsRNA-thanatin_F	GACTTCATCAAGATGCATGTTG
dsRNA-thanatin_R	AGTAAATTATCGGTACTCTCCC
Target-T7_F	TAATACGACTCACTATAGGG GATCTACTAGTCATATGGAT
Target-T7_R	TAATACGACTCACTATAGGG GACGGCCAGTGAAT
β-Lactam_T7_F	TAATACGACTCACTATAGGG CTATGTGGCGCGGTATTAT
β-Lactam_T7_R	TAATACGACTCACTATAGGG CAGAAGTGGTCCTGCAACT

### Measurement of Symbiont Titers in the M4 Region

Bacterial cells were washed with 10 mM PB and suspended in Grace’s Insect Medium (Gibco, United States) to 1.0 × 10^7^ cells/ml bacterial solutions (Kim et al., 2013a; Lee et al., 2020). Briefly, two microliters of *thanatins* dsRNA were systemically injected into 3-day-old *R. pedestris* male adults for immune suppression. In addition, two microliters of the bacterial cell suspensions were injected into the joint of the hind leg that connects to the thorax of the insect. *E. coli* K12 cells were injected 24 h after dsRNA injection. Individual M4 crypts dissected from *R. pedestris* 3 days after inoculation were collected in 100 μl of PB, homogenized using a plastic pestle, and serially diluted with PB. The diluted samples were spread onto YG-RIF agar plates and colonies grown at 30°C were counted 2 days later. The symbiont titers for each insect were evaluated. The survival rate was evaluated 7 days later after the bacterial septic injections.

### Identification of Symbiotic *Burkholderia* Cells in Host Hemolymph

To detect gut-colonized symbionts penetrating the M4 crypt into the host hemolymph, each *R. pedestris* insect with its legs removed was immersed in 200 μl of 10 mM PB (Kim et al., 2015b). After homogenization via vortexing, the insects were removed, and the buffer was serially diluted to measure CFUs. The diluted samples were spread onto YG-RIF agar plates. Colonies grown at 30°C were counted 2 days later.

### Tree Analysis

The protein sequences of the thanatin isoforms from *R. pedestris* were compared. The protein sequences were aligned with ClustalW using MegAlign (ver. 7.1.0.). Phylogenetic trees for the alignment were calculated via the neighbor-joining method and the Jones–Taylor–Thornton model. The bootstrap tree was obtained from the protein sequence using the Molecular Evolutionary Genetic Analysis-X software.

### Statistical Analysis

The statistical significance of data was determined by unpaired Student’s *t*-test or one-way ANOVA with Tukey’s correction using SPSS 23 and GraphPad Prism 8 software.

## Results

### Thanatin Isoforms With Repeated Specific Regions

**FIGURE 1:**
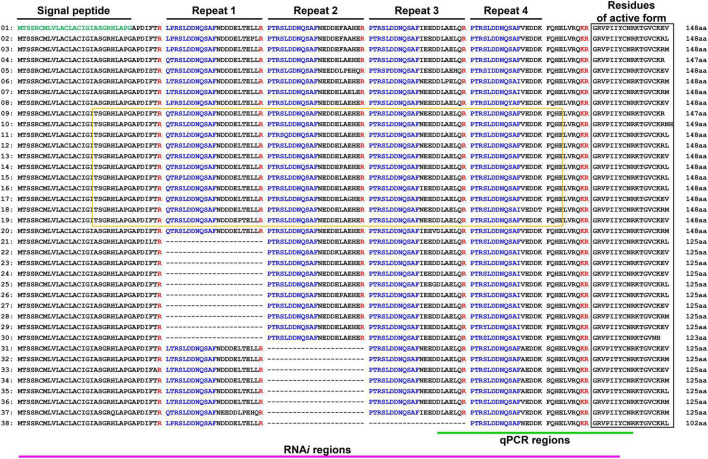
Multiple precursor proteins of thanatin isoforms in *R. pedestris*. Structure analysis of precursor molecules (green: signal peptide region; blue: repetitive sequence). Black and yellow boxes indicate the region of antimicrobial activity and MACPF domain.

First, to identify thanatin isoforms in *R. pedestris*, total RNA was collected from insect fat body and cDNA was synthesized by RACE-PCR. We cloned putative target DNA fragments encoding precursor proteins of thanatin isoforms, ranging from 125 to 148 residues in length. The InterPro protein families database^1^ was used to identify the functional domain of the thanatin isoforms. Thanatin precursor proteins contain a signal peptide at the N-terminus. Four repetitive sequences cleaved by trypsin, which did not contain any conserved motifs, were identified (Figure 1).

### Three Active Forms of Thanatin Isoforms

**FIGURE 2:**
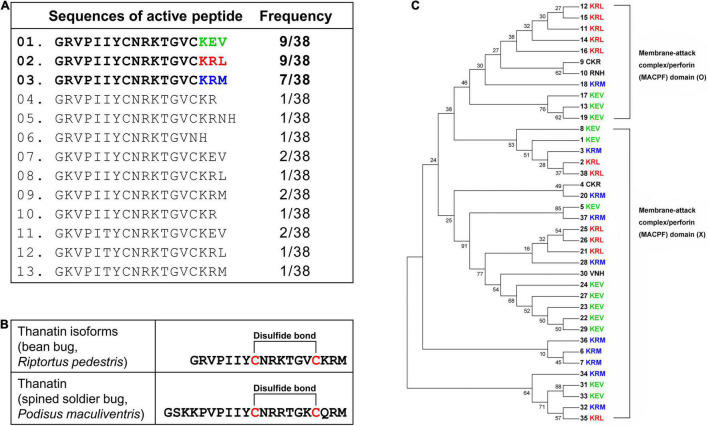
Alignment of active thanatin isoforms. **(A)** The alignment of amino acid sequences and frequency of thanatin isoforms. **(B)** Comparison of amino acid sequences and structure between thanatin KRM isoform from *R. pedestris* and thanatin of *P. maculiventris*. The line between cysteine residues represents a disulfide bond. **(C)** Phylogenetic tree using precursor sequences of thanatin isoforms. Thanatin precursor proteins with (upper clade, O) or without (bottom clade, X) the membrane attack complex/perforin (MACPF) domain.

Structurally active thanatin isoforms derived from 38 precursor proteins were aligned, and their frequencies were investigated. The C-terminus region had three main active forms: KEV, KRL, and KRM (Figure 2A). When thanatin isoforms in *R. pedestris* were compared with those of *P. maculiventris*, their active sequences showed similarities and harbored a conserved cysteine disulfide bridge (Figure 2B). The phylogenetic analysis showed that thanatin isoforms can be categorized into two groups: with (upper clade, O) or without (bottom clade, X) the membrane attack complex/perforin (MACPF) domain (Figure 2C).

### Antimicrobial Activity of Thanatin KEV Isoform Against Gut Colonized Symbionts

**FIGURE 3:**
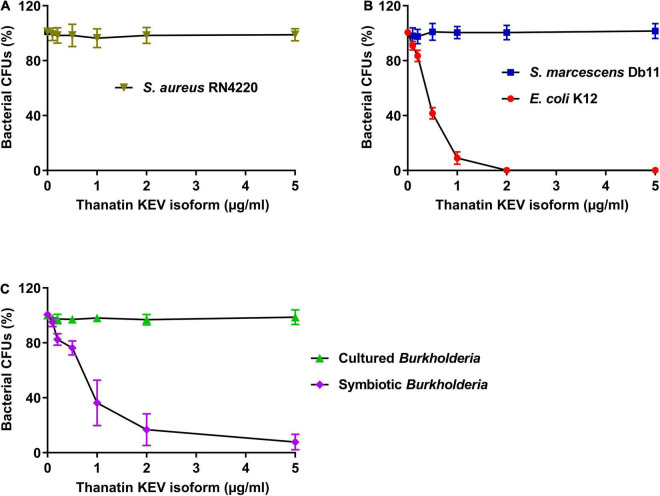
Measurement of the antibacterial activity of thanatin isoforms. The antibacterial activity of thanatin isoforms against **(A)** *S. aureus* RN4220, **(B)** *E. coli* K12 and *S. marcescens* Db11, **(C)** cultured *Burkholderia* and symbiotic *Burkholderia* cells. The CFUs were calculated and normalized using AMP-untreated CFUs set to 100%. Data are expressed as mean ± SD (*n* = 3) and are representative of three independent experiments.

To evaluate the antimicrobial activity of the thanatin KEV isoform as an AMP in *R. pedestris*, the antibacterial activities against *E. coli* K12, *S. marcescens* Db11, *S. aureus* RN4220, cultured *Burkholderia*, and symbiotic *Burkholderia* were measured. The *Staphylococcus* strain, a gram-positive bacterium, had high resistance to the thanatin KEV isoform (Figure 3A). When the KEV isoform was incubated with gram-negative bacterial cells, the growth of the *E. coli* cells was markedly reduced, but *S. marcescens* was not killed by the thanatin KEV isoform (Figure 3B). In contrast to *in vitro*-cultured *Burkholderia* cells, *in vivo*-colonized symbionts living in the M4 crypt of *R. pedestris* were highly susceptible to the KEV isoform (Figure 3C). Other main isoforms showed similar antibacterial activity to the KEV isoform (Supplementary Figure S1).

### Modulation of Thanatin Isoforms by *Burkholderia* Gut Symbiont

**FIGURE 4:**
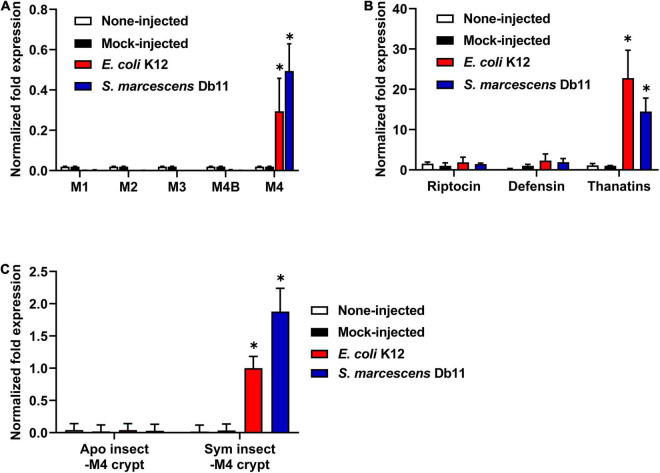
The expression levels of *thanatin* genes in *R. pedestris*. **(A)** mRNA expression levels of *thanatin* genes in each midgut region (M1, M2, M3, M4B, and M4) of the Sym-male insect upon gram-negative *E. coli* K12 and *S. marcescens* Db11 injection. **(B)** mRNA expression levels of three AMP genes in the M4 crypt of the Sym-male insect upon gram-negative *E. coli* K12 and *S. marcescens* Db11 injection. **(C)** mRNA expression levels of *thanatin* genes in the M4 crypt of both the Apo-insect and Sym-male insect upon gram-negative *E. coli* K12 and *S. marcescens* Db11 injection. The ampicillin resistance gene (*amp*) was used as a mock-control in the RNA*i* experiment. The expression levels of the *thanatin* genes were normalized to the expression level of **(A,C)** *E. coli* K12-injected insect or **(B)** mock-control, which was set as 1. Error bars indicate the standard deviation of the mean (*n* = 6). Asterisk indicates a significant difference between each group (*p < 0.01; unpaired *t*-test). (**A**; M1, M2, M3, and M4B vs. M4), (**B**; Riptocin or Defensin vs. Thanatins), and (**C**; Apo insect-M4 crypt vs. Sym insect-M4 crypt).

Based on the above results, thanatin isoforms could be closely involved in the control of symbiotic bacteria colonization as well as the defense response of insects against invasive pathogens. To investigate the expression of thanatin isoforms against systemic bacterial injection in insect midgut regions, total RNA was extracted from Sym-male insect, and the target DNA fragments were amplified with gene-specific primers (Table 2). Interestingly, the expression levels of *thanatin* genes were comparatively high in the M4 crypt, where the symbiotic bacteria reside, whereas it was not expressed in the other midgut regions of M1 to M4B (Figure 4A). Further, the expression of *thanatin* genes was not detected without immune-challenge despite the presence of *Burkholderia* gut symbionts in the M4 crypt (mock-injected and non-injected; Figure 4A). To determine whether *riptocin* and *defensin* genes, which are other AMPs of *R. pedestris*, were also expressed in the symbiotic organ M4 crypt, their expression was evaluated. The expression of *thanatin* genes after a bacterial challenge with *E. coli* or *S. marcescens* cells was higher than that of the *riptocin* and *defensin* genes (Figure 4B). We also measured the mRNA levels of the *thanatin* genes in the M4 crypt of Apo-*R. pedestris* without symbionts. Expression of thanatin isoforms in Apo-*R. pedestris* was not detected despite induction of immune response by systemic *E. coli* or *S. marcescens* injection (Figure 4C). Taken together, these results demonstrate that the *Burkholderia* gut symbiont modulates the expression of thanatin isoforms.

### Control of Gut Symbiont Population by Thanatin Isoforms During Systemic Bacterial Injection

**FIGURE 5:**
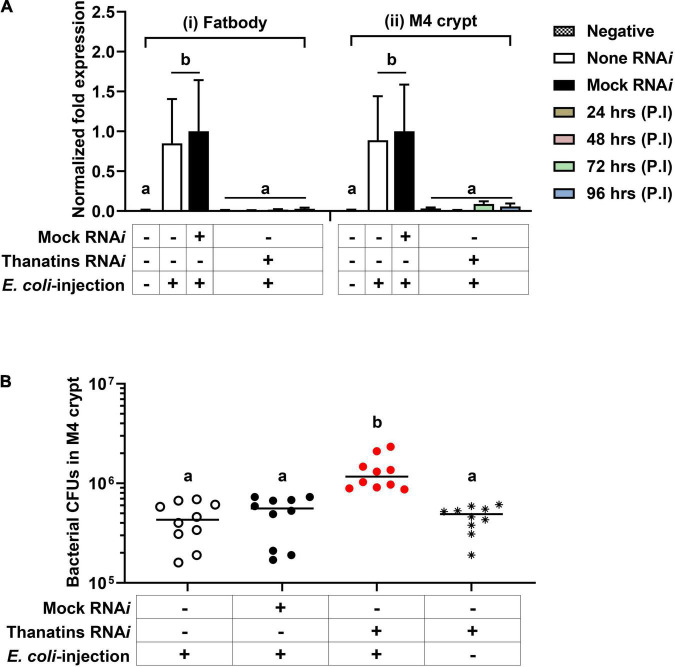
The population of *Burkholderia* gut symbiont after RNA*i* of the thanatin isoforms. **(A)** mRNA expression levels of *thanatin* genes in (i) the fat body and (ii) M4 crypt after RNA*i* of the target gene upon systemic *E. coli* K12 injection. The expression levels of the *thanatin* genes were normalized to the expression level of each mock-control, which was set as 1. Data are expressed as mean ± SD (*n* = 6). Different letters (a and b) on the top of the columns indicate statistically significant differences (*P* < 0.05; one-way ANOVA with Tukey’s correction). **(B)** The population of M4 crypt-colonized *Burkholderia* cells in the *thanatin* genes silenced *R. pedestris* upon systemic *E. coli* K12 injection. Data are expressed as mean ± SD (*n* = 10). Different letters (a and b) on the top of the columns indicate statistically significant differences (*P* < 0.05; one-way ANOVA with Tukey’s correction). The CFUs in M4 crypts were measured on 4 days after dsRNA injection or 3 days after *E. coli* K12 cells injection.

Based on the unique expression of the thanatin isoforms in Sym-*R. pedestris*, we hypothesized that thanatin isoforms may play another role in the M4 crypt. To elucidate the interaction between the *Burkholderia* gut symbiont and thanatin isoforms in the M4 crypt, the expression of *thanatin* genes was suppressed by RNA*i*. The expression level of the *thanatin* genes was completely reduced from 1 day after injection of *thanatin* dsRNA in both fat body and M4 crypts (Figure 5A). Changes in symbiotic bacterial growth were monitored in the M4 crypt following systemic *E. coli* K12 injection. Interestingly, the suppression of *thanatin* genes significantly increased the population of *Burkholderia* gut symbionts (Figure 5B).

### Insecticidal Activity and Growth of Gut Symbiont in the Hemolymph of Host Insect

**FIGURE 6:**
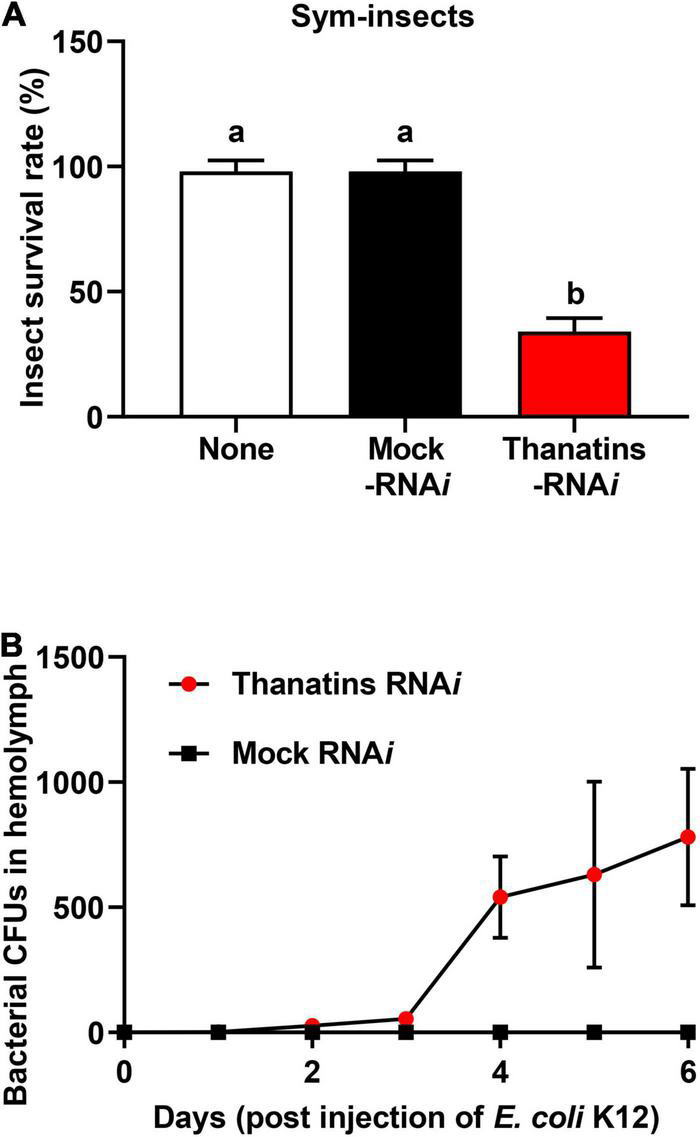
Insecticidal effect of *Burkholderia* gut symbionts in the hemolymph during systemic *E. coli* K12 injection. **(A)** Survival rates of the *thanatin* gene-silenced Sym-male insects upon systemic *E. coli* K12 injection. Data are expressed as mean ± SD (*n* = 10). Different letters (a and b) on the top of the columns indicate statistically significant differences (*P* < 0.05; one-way ANOVA with Tukey’s correction). **(B)** Comparison of the symbiont cells detected in insect’s hemolymph by RNA*i* and systemic *E. coli* K12 injection. Data are expressed as mean ± SD (*n* = 3). All data are representative of three independent experiments.

**FIGURE 7:**
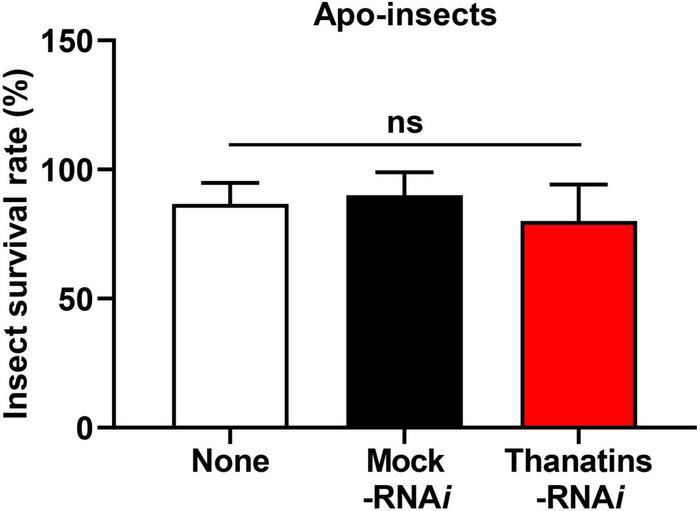
Survival rates of the *thanatin* gene-silenced Apo-insect upon systemic *E. coli* K12 injection. Data are expressed as mean ± SD (*n* = 10). Data are representative of three independent experiments (ns = non-significant) (*P* < 0.05; one-way ANOVA with Tukey’s correction).

We evaluated the health parameters of host insect to determine the impacts on *R. pedestris* that can be caused by an increase in the symbiont population in the M4 crypts. Briefly, the survival rate of *thanatins* knock-downs Sym-*R. pedestris* was examined after systemic injection with *E. coli* cells. In addition, to prevent epithelial damage of M4 crypts by systemically injected *E. coli* cells, an appropriate 10^4^ CFUs was used in the experiment to induce an insect immune response but not cause any harm to the insects. The survival rate of *thanatins*-suppressed Sym-*R. pedestris* was found to be markedly lower than that in the control group under the context of systemic injection with *E. coli* cells (Figure 6A). In contrast, the survival rate of *thanatins*-suppressed Apo-*R. pedestris* did not show any statistically significant differences (Figure 7). These results suggest that overgrown symbiont in the M4 crypts by co-injection with RNA*i* and *E. coli* cells adversely affect the survival of host insects. Since systemically injected *E. coli* cells would have been cleared by riptocin AMPs in the hemolymph of insects, it can be expected that overgrown *Burkholderia* cells are directly linked to insect survival. Based on these results, we investigated whether overgrown symbiont cells were observed in the hemolymph. *Burkholderia* gut symbiont cells were found to grow rapidly in the hemolymph 3 days post-injection (Figure 6B). Taken together, when the gut symbiont grows out of control in the host, survival of the host decreases, while at the same time gut symbionts are observed in the host’s hemolymph.

## Discussion

Here, we revealed that 38 thanatin precursor proteins consisted of three major active forms. In addition, expression levels of thanatin genes were significantly increased in the M4 crypt of symbiotic insects upon systemic bacterial injection. Moreover, synthetic thanatin isoforms exhibited antibacterial activity against gut-colonized *Burkholderia* symbionts rather than *in vitro*-cultured *Burkholderia* cells. Interestingly, the suppression of *thanatin* genes significantly increased the population of *Burkholderia* gut symbionts in the M4 crypt under systemic *Escherichia coli* K12 injection. Finally, overgrown *Burkholderia* gut symbionts were observed in the hemolymph of host insects and exhibited insecticidal activity.

As thanatin peptide was first identified in *P. maculiventris* insects, research has mainly focused on the differences in antimicrobial activity based on the structure of thanatin (Taguchi et al., 2000; Sinha et al., 2017; Vetterli et al., 2018). Thanatin peptides contain a disulfide bond between Cys11 and Cys18 at their C-terminus (Bulet et al., 1999). The core structure of thanatin is an anti-parallel β-sheet structure from Ile8 to the C-terminus and is maintained by a single disulfide bond; the seven amino acids at the N-terminus form the arm structure (Fehlbaum et al., 1996). Three residues (QRM) at the C-terminus are important for its antibacterial activity, and three residues (GSK) at the N-terminal region are required for its antifungal activity (Powers and Hancock, 2003; Bulet and Stocklin, 2005). The number of amino acid residues within a single disulfide bond affects antibacterial activity (Lee et al., 2002). Collectively, the C-terminal residues of thanatin are more critical for antimicrobial activity than the N-terminal residues (Dash and Bhattacharjya, 2021). Despite studies on the structure and antimicrobial activity of thanatin, the precursor molecules of this peptide have not been identified. Therefore, we report for the first time the amino acid sequences of the thanatin precursor proteins and demonstrate the novel role of the peptide thanatin in the insect–symbiont interaction.

The innate immune mechanisms in the hemimetabolous insect, *R. pedestris*, have been studied markedly less than those in holometabolous insects. However, recent research on the immune response has been steadily progressing owing to its advantages as a symbiotic model (Kim et al., 2015a; Jang et al., 2017; Lee D. J. et al., 2017). Although the three AMPs, riptocin (pyrrhocoricin-like), defensin, and thanatin, are well known in insects (Kragol et al., 2001; Oppenheim et al., 2003; Dash and Bhattacharjya, 2021), their molecular mechanisms and physiological roles in *R. pedestris–B. insecticola* symbiosis are still unclear. The peptide riptocin is derived from a precursor protein consisting of 678 amino acid residues with 14 tandem repeats (Miura et al., 1996). Each repeat contains a proline-rich region, which is also found in other insects (Levashina et al., 1995; Li et al., 2014; Taniguchi et al., 2016). While riptocin is produced from a single multipeptide precursor, we revealed that *R. pedestris* insects have 38 genes that can produce 13 different types of thanatin isoforms (Figure 1). The multiple precursor structures of thanatin isoforms are unique in *R. pedestris* and were categorized into two groups depending on the existence of the MACPF domain (Figure 2C). The MACPF domain is found in the complement system of mammalian immunity, and molecules with the MACPF domain are known to play important roles in defense against bacterial and viral infections (Rosado et al., 2008; Reboul et al., 2016; Liu and Lieberman, 2020). In the insect model, the MACPF domain was identified only in the *Drosophila* Torso-like protein, which is involved in the control of development and cellular immune response (Johnson et al., 2013; Forbes-Beadle et al., 2016).

In this study, thanatin isoforms showed antibacterial activities against *E. coli* K12 and gut colonized symbiont, but not against *S. marcescens* Db11 and cultured *Burkholderia* cells (Figure 3). This difference in antibacterial activity could be due to the molecular interaction between the thanatin isoforms and the lipopolysaccharide (LPS) pattern of each bacterium. Thanatin causes charge neutralization of the outer membrane, displacing the stabilizing Ca^2+^ ions from the LPS molecules (Ma et al., 2019). The charge-neutralized outer membrane efficiently induces cell aggregation, eventually leading to bacterial cell death (Wu et al., 2010; Sinha et al., 2017). In addition, thanatin binds to the LPS transport protein complex (Lpt proteins) located between the inner and outer membranes and inactivates metallo-β-lactamase in the periplasm (Vetterli et al., 2018; Moura et al., 2020). *S. marcescens* Db11 and cultured *Burkholderia* have a smooth type of LPS with lipid A plus core oligosaccharide (OS) and *O*-antigen, whereas *E. coli* K12 and gut colonized symbionts have a rough type of LPS lacking the *O*-antigen (Washizaki et al., 2016; Ebbensgaard et al., 2018). Of note, the main isoforms of thanatin exhibit selective antibacterial activity depending on growth conditions (Figure 3C) or the existence of *O*-antigen in the LPS layer (Figure 3B). Therefore, the structural differences in the LPS layers may affect the antibacterial activity of thanatin isoforms.

In many insects, mutualistic bacteria can inhabit symbiotic organs, or bacteriomes, providing nutrients to the host or enhancing the immune response, among other potential functions (Moran et al., 2003; Douglas, 2014; Flórez et al., 2015). Interestingly, the overgrown *Burkholderia* gut symbionts are eliminated by the host immune response (Kim et al., 2013a; Byeon et al., 2015), but can move to the hemolymph from the M4 crypt by immune suppression, such as the inhibition of thanatin isoforms with *E. coli* K12 injection (Figure 6B). The regulation of symbiont populations by host insects has been shown to influence their migration. A previous report revealed that the coleoptericin-A AMP of weevil insects selectively targets endosymbionts within the bacteriocytes and regulates their growth by inhibiting cell division (Anselme et al., 2008; Login et al., 2011). Therefore, similar to the AMP, coleoptericin-A, the thanatin isoforms of *R. pedestris* may also play an important role in regulating and maintaining colonization of the *Burkholderia* gut symbiont.

Recently, we reported that the cell wall components of *Burkholderia* gut symbionts are important factors for successful symbiosis (Kim et al., 2013b,2016b,^2017^). As mentioned above, the symbiotic *Burkholderia* colonized in the M4 crypt of *R. pedestris* lacks an *O*-antigen residue (Kim et al., 2015b). However, it has not yet been elucidated what additional changes occurred in the symbiotic *Burkholderia* cells other than the *O*-antigen deficiency. A further study revealed that the colonization of *Burkholderia* gut symbionts with truncated core OSs impairs host health and reduces insect survival during systemic bacterial injection (Kim et al., 2017). Based on previously reported studies that LPS composed of lipid A and core OS residues and *O*-antigens are associated with bacterial endotoxin (Raetz and Whitfield, 2002; Nikaido, 2003), these results suggest that *Burkholderia* gut symbionts that migrate to the hemolymph owing to overpopulation can kill insects. When Sym-*R. pedestris* was injected with *Burkholderia* LPS mutant strains, the insect survival rate was significantly decreased (Supplementary Figure S2). In addition, *Burkholderia* gut symbionts, which moved from the M4 crypt, showed insecticidal activity when co-injected with *E. coli* and impaired thanatin production in the insect (Figure 6A). However, insecticidal activity was not observed in *thanatins*-suppressed Apo-*R. pedestris* (Figure 7). This result suggests that systematically injected *E. coli* cells did not cause any harm to Apo-*R. pedestris* insects without *Burkholderia* gut symbionts. Therefore, the hypothesis that *Burkholderia* symbionts came out into hemolymph due to damage to midgut epithelial cells caused by systemically injected *E. coli* cells can be excluded. Taken together, the *Burkholderia* gut symbionts, when maintained up to a suitable number of populations in the symbiotic organ, confer positive effects, such as immunity enhancement on the host insect, but may exhibit an insecticidal effect in the hemolymph of *R. pedestris*, when the host cannot control them and under the context of a systemic injection with *E. coli* cells.

**FIGURE 8:**
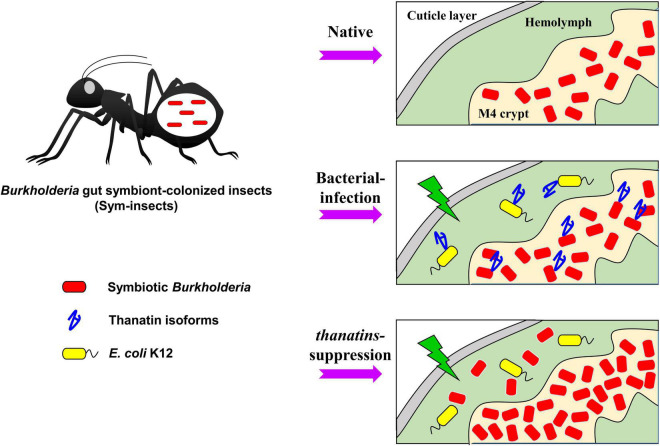
Putative mechanism of action of the thanatin isoforms against *Burkholderia* gut symbionts during systemic bacterial injection. Thanatin isoforms maintain the gut bacterial population in the M4 crypt during systemic bacterial injection. In contrast, in absence of thanatin (*thanatins* RNA*i*) and after systemic *E. coli* K12 injection, overgrown *Burkholderia* gut symbionts were observed in the hemolymph of the *R. pedestris* host and exhibited insecticidal activity.

The *Burkholderia* gut symbionts accumulate polyhydroxyalkanoate (PHA) granules, a bacterial endocellular storage polymer, to adapt to the harsh environment in the midgut region of *R. pedestris* (Kim et al., 2013c). PHA granules allow the *Burkholderia* gut symbiont to resist nutrient depletion and environmental stress. Given the various resistance capabilities provided by PHA granules to bacterial cells (Anderson and Dawes, 1990; Kadouri et al., 2003; Ratcliff et al., 2008), the gut symbionts may also become resistant to insect immune cells due to the PHA granules. Therefore, we carefully suggest the following hypothetical mechanisms (Figure 8): in the natural state, *R. pedestris* synthesize thanatin isoforms during systemic injection of foreign bacteria to properly regulate the growth of *Burkholderia* gut symbionts. When thanatin genes were suppressed, the *Burkholderia* population in the M4 crypt was increased and subsequently migrated to the hemolymph through the intestinal barrier. Although migrating *Burkholderia* gut symbionts are engulfed by immune cells, such as phagocytic hemocytes in the hemolymph, these gut bacterial cells may become resistant to cellular immune responses by their own PHA granules. Eventually, *Burkholderia* gut symbionts with truncated core OSs present in the hemolymph can impair host health and reduces insect survival.

In this study, scientific evidence is needed as to whether symbiotic bacteria are resistant to hemocytes by PHA granules and what endotoxin substances they secrete. Further studies could clearly demonstrate that the *Burkholderia* gut symbionts outside the M4 crypt reduces the immunity of the *R. pedestris* host insect.

## Conclusion

Thanatin isoforms are derived from multiple precursor proteins and function as AMPs that modulate the population of gut symbionts. This study provides novel information on the role of thanatin isoforms in the symbiotic organs of *R. pedestris* and the identification of novel symbiotic factors.

In addition, this study reveals very interesting findings about the indirect interaction between resident symbionts and foreign infecting bacteria that are modulated by the host immune system. Finally, this study can be considered as basic data on how stability can be maintained after establishment of symbiosis.

## Data Availability Statement

The original contributions presented in the study are included in the article/Supplementary Material, further inquiries can be directed to the corresponding author/s.

## Ethics Statement

The animal study was reviewed and approved by all animal treatment guidelines applicable to invertebrate animals under international and South Korean law have been followed.

## Author Contributions

JL and D-WL conceived and designed the study and wrote the manuscript. JL performed the experiments. JL and WHC analyzed the data. All the authors read and approved the final manuscript.

## Conflict of Interest

The authors declare that the research was conducted in the absence of any commercial or financial relationships that could be construed as a potential conflict of interest.

## Publisher’s Note

All claims expressed in this article are solely those of the authors and do not necessarily represent those of their affiliated organizations, or those of the publisher, the editors and the reviewers. Any product that may be evaluated in this article, or claim that may be made by its manufacturer, is not guaranteed or endorsed by the publisher.

## Footnotes

1
https://www.ebi.ac.uk/interpro/

## Supplementary Material

Supplementary Figure S1 The antibacterial activity of **(i)** thanatin KRL isoform and **(ii)** thanatin KRM isoform against **(A)** *E. coli* K12 and *S. marcescens* Db11, **(B)** Cultured *Burkholderia* and symbiotic *Burkholderia* cells. CFUs were normalized by AMP-untreated CFUs set to 100%. Data are expressed as mean ± SD (*n* = 3). Data are representative of three independent experiments.

Supplementary Figure S2 Survival rates of *R. pedestris* upon systemic injection with the LPS mutant strains. Data are expressed as mean ± SD (*n* = 10). Data are representative of three independent experiments.
