# Supplementary figures and images for: Multiple Precursor Proteins of Thanatin Isoforms, an Antimicrobial Peptide Associated With the Gut Symbiont of Riptortus pedestris
Source: Front Microbiol. 2022 Jan 5;12:796548. doi: 10.3389/fmicb.2021.796548 (PMC8767025; doi:10.3389/fmicb.2021.796548)

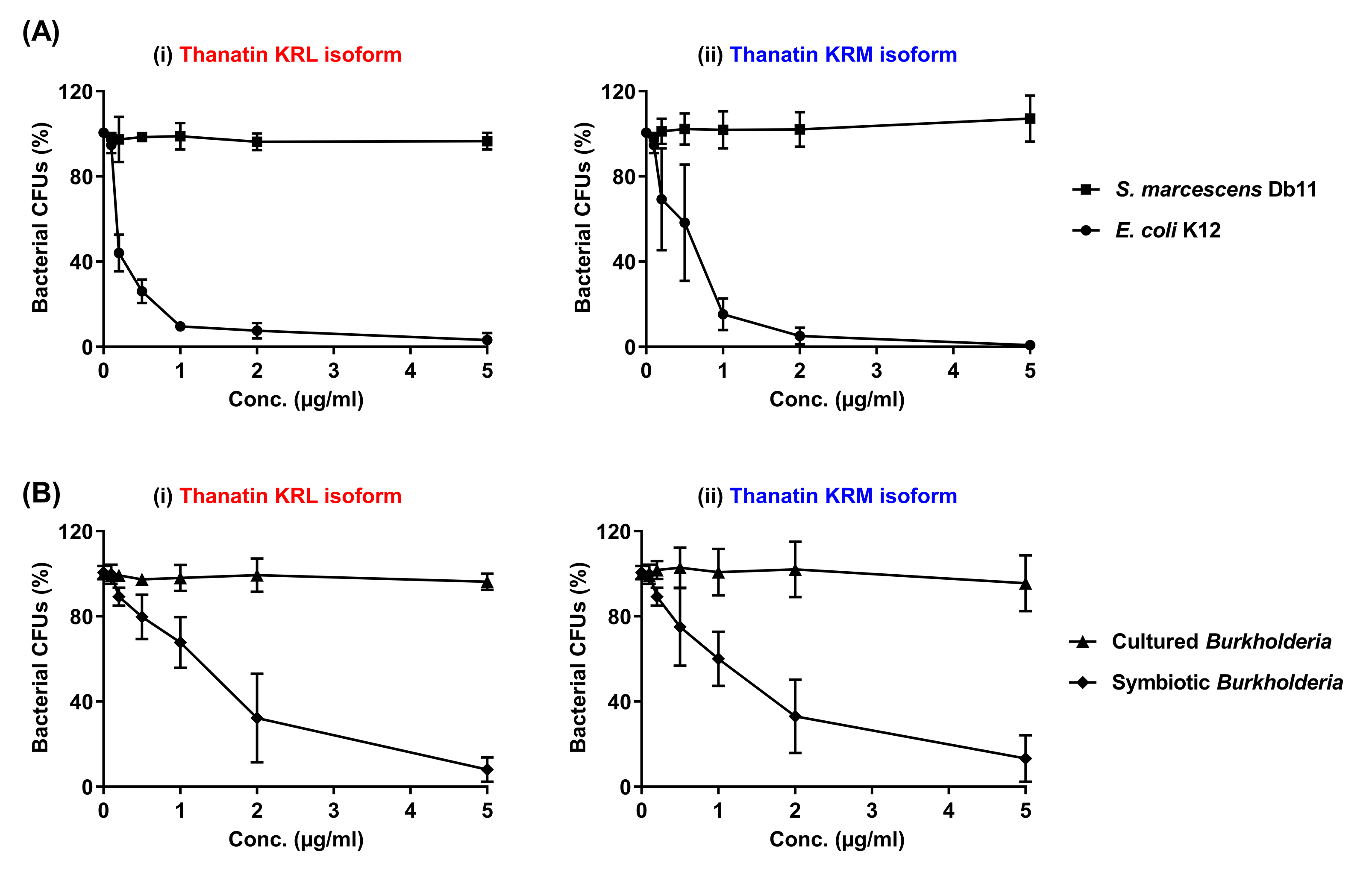

Supplement: Supplementary Figure S1 — The antibacterial activity of (i) thanatin KRL isoform and (ii) thanatin KRM isoform against (A) E. coli K12 and S. marcescens Db11, (B) Cultured Burkholderia and symbiotic Burkholderia cells. CFUs were normalized by AMP-untreated CFUs set to 100%. Data are expressed as mean ± SD (n = 3). Data are representative of three independent experiments. [file Image_1.JPEG]

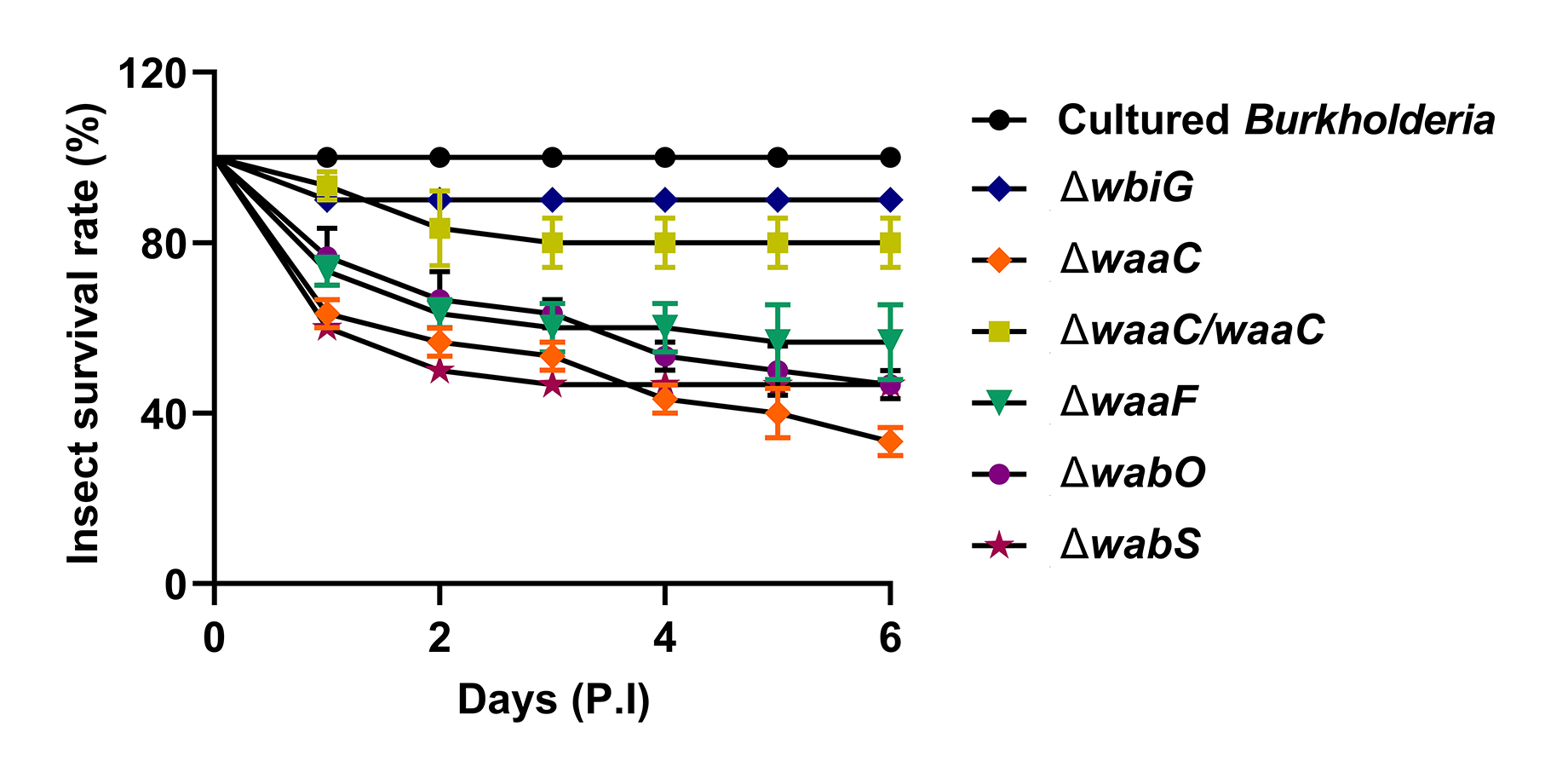

Supplement: Supplementary Figure S2 — Survival rates of R. pedestris upon systemic injection with the LPS mutant strains. Data are expressed as mean ± SD (n = 10). Data are representative of three independent experiments. [file Image_2.JPEG]
